# Energy Metabolism Is Altered in Radioresistant Rectal Cancer

**DOI:** 10.3390/ijms24087082

**Published:** 2023-04-11

**Authors:** Croí E. Buckley, Xiaofei Yin, Sebastian Meltzer, Anne Hansen Ree, Kathrine Røe Redalen, Lorraine Brennan, Jacintha O’Sullivan, Niamh Lynam-Lennon

**Affiliations:** 1 Department of Surgery, School of Medicine, Trinity Translational Medicine Institute, Trinity St James’s Cancer Institute, Trinity College Dublin, D08 NHY1 Dublin, Ireland; 2 UCD School of Agriculture and Food Science, UCD Institute of Food and Health, Conway Institute, University College Dublin, D04 V1W8 Dublin, Ireland; 3 Department of Oncology, Akershus University Hospital, 1478 Lørenskog, Norway; 4 Department of Physics, Norwegian University of Science and Technology, 7491 Trondheim, Norway

**Keywords:** rectal cancer, radioresistance, metabolism, oxidative phosphorylation, glycolysis, metabolites

## Abstract

Resistance to neoadjuvant chemoradiation therapy is a significant clinical challenge in the management of rectal cancer. There is an unmet need to identify the underlying mechanisms of treatment resistance to enable the development of biomarkers predictive of response and novel treatment strategies to improve therapeutic response. In this study, an in vitro model of inherently radioresistant rectal cancer was identified and characterized to identify mechanisms underlying radioresistance in rectal cancer. Transcriptomic and functional analysis demonstrated significant alterations in multiple molecular pathways, including the cell cycle, DNA repair efficiency and upregulation of oxidative phosphorylation-related genes in radioresistant SW837 rectal cancer cells. Real-time metabolic profiling demonstrated decreased reliance on glycolysis and enhanced mitochondrial spare respiratory capacity in radioresistant SW837 cells when compared to radiosensitive HCT116 cells. Metabolomic profiling of pre-treatment serum samples from rectal cancer patients (*n* = 52) identified 16 metabolites significantly associated with subsequent pathological response to neoadjuvant chemoradiation therapy. Thirteen of these metabolites were also significantly associated with overall survival. This study demonstrates, for the first time, a role for metabolic reprograming in the radioresistance of rectal cancer in vitro and highlights a potential role for altered metabolites as novel circulating predictive markers of treatment response in rectal cancer patients.

## 1. Introduction

Colorectal cancer (CRC) is the third most commonly diagnosed cancer worldwide, accounting for an estimated 10% of cancers [1]. CRC also has the second-highest rate of cancer-related mortality, resulting in an estimated 9.4% of cancer-related deaths [1]. One third of all CRCs occur in the lower bowel, or the rectum, and it is predicted that in Ireland incidence rates of rectal cancer will rise by 93–97% by the year 2045 [2]. The standard of care for locally advanced rectal cancer is neoadjuvant chemoradiation therapy (neoCRT) followed by surgery [3]. Typically, patients receive long-course radiotherapy (45–50 Gy in 1.8–2 Gy fractions over 5–6 weeks) combined with a 5-fluorouracil (5-FU)- or capecitabine-based chemotherapy regimen prior to surgery [4]. A pathological complete response (pCR), which is characterized by no viable tumor cells, is a known predictor of overall and disease-free survival [5]. Unfortunately, pCR rates remain low at <30% [5]. Radiation plays a central role in the treatment of rectal cancer; however, resistance to treatment is a major clinical challenge in the management of rectal cancer [6]. Consequently, there is an unmet need to identify the mechanisms underlying therapeutic resistance to identify biomarkers predicting treatment response for improved patient stratification and novel therapeutic targets to enhance patient response to the standard of care, to improve treatment and survival for rectal cancer patients.

It has long been established that intrinsic tumor factors, such as tumor volume, stage and hypoxia, contribute to patient response to radiation therapy. However, these factors alone do not determine radioresponse, suggesting that altered molecular mechanisms and biological pathways also contribute to radioresistance [7]. The exact mechanisms contributing to radioresistance in rectal cancer are poorly understood [8]. Furthermore, research investigating mechanisms of radioresistance in rectal cancer is limited by the poor availability of characterized in vitro models of rectal cancer, with most research utilizing models of colonic origin [9,10]. Importantly, as colon and rectal cancers are demonstrated to be distinct diseases, and as radiation therapy is a common treatment in the management of rectal cancer but not colon cancer [11,12,13,14], the identification and characterization of in vitro models of radioresistant rectal cancer are crucial to elucidate the mechanisms of radioresistance in rectal cancer.

The aim of this study was to investigate the mechanisms underlying radioresistance in rectal cancer using both an in vitro model of inherently radioresistant rectal cancer and pre-treatment sera from rectal cancer patients. This study demonstrates that radioresistant rectal cancer cells have altered cell cycle distribution, cell cycle progression and enhanced DNA damage repair. Furthermore, we demonstrate through live-cell metabolic profiling and upstream transcriptomic analysis that radioresistant rectal cancer cells have altered metabolism, specifically a decreased reliance on glycolysis. We also demonstrate that the metabolome of pre-treatment sera from rectal cancer patients is significantly altered in patients having a subsequent poor response to neoCRT and poorer survival, supporting the role of altered metabolism in treatment response and highlighting the potential for metabolites as circulating biomarkers in predicting response to therapy in rectal cancer patients.

## 2. Results

### 2.1. Identification of an In Vitro Model of Radioresistant Rectal Cancer

#### Clonogenic Survival of SW837 and HCT116 Cells Following X-ray Radiation and 5-Fluorouracil (5-FU) Chemotherapy

**Figure 1:**
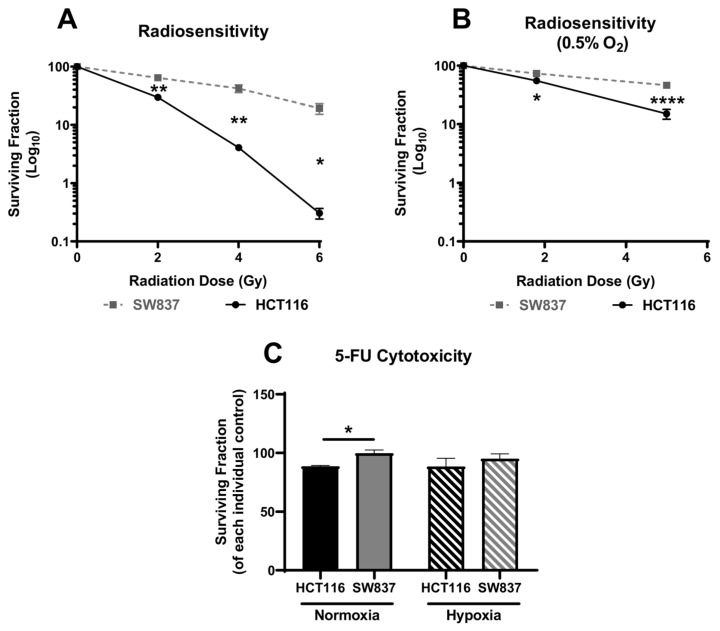
Identification of an in vitro model of inherently radioresistant/radiosensitive CRC. Clonogenic survival of HCT116 and SW837 cells treated with (**A**) X-ray radiation (2 Gy, 4 Gy or 6 Gy) under normoxia or (**B**) X-ray radiation (1.8 Gy or 5 Gy) under hypoxia or (**C**) 5-FU (15 µM, 30 h) or dimethyl sulfoxide (DMSO) vehicle control under normoxic or hypoxic conditions relative to controls, as assessed by the gold-standard clonogenic assay. Data are presented as means ± SEMs for at least three independent experiments. Statistical analysis was performed by *t*-testing or ANOVA, as appropriate. * *p* < 0.05, ** *p* < 0.01, **** *p* < 0.0001.

To identify an in vitro model of inherent radioresistance in rectal cancer, the basal sensitivity of SW837 rectal adenocarcinoma cells to X-ray radiation was assessed using the gold-standard clonogenic assay and compared to the well-characterized, radiosensitive HCT116 colon cancer cell line. SW837 rectal cancer cells were demonstrated to be significantly more resistant to fractionated doses of 2, 4 and 6 Gray (Gy) X-ray radiation when compared to HCT116 cells (*p* = 0.009, *p* = 0.004 and *p* = 0.01, respectively; Figure 1A). The mean surviving fractions of radioresistant SW837 cells following exposure to 2, 4 and 6 Gy were 64.23%, 42.18% and 19.3%, respectively. In contrast, the mean surviving fractions of radiosensitive HCT116 cells following exposure to 2, 4 and 6 Gy were 29.83%, 4.09% and 0.31%, respectively.

As a hypoxic environment is known to contribute to radioresistance, the effect of hypoxia on the inherent radiosensitivity of HCT116 and SW837 cells was assessed. Following exposure to a clinically relevant dose of 1.8 Gy radiation under hypoxia (0.5% O_2_), SW837 rectal cancer cells were significantly more resistant to X-ray radiation when compared to HCT116 colon cancer cells (*p* = 0.0284) (Figure 1B). (Mean surviving fractions following 1.8 Gy under hypoxia: in HCT116 cells: 55.38%; in SW837 cells: 73.34%.) Furthermore, following 5 Gy irradiation, SW837 cells remained more resistant to radiation when compared to HCT116 cells (*p* = 0.0009) (Figure 1B). (Mean surviving fraction following 5 Gy under hypoxia: in HCT116 cells: 15.07%; in SW837 cells: 46.34%.) Importantly, these data demonstrate that HCT116 and SW837 cells are robust in vitro models of radiosensitive and radioresistant CRC under both normoxic and hypoxic conditions.

To determine whether the resistance of SW837 cells was specific to radiation, HCT116 and SW837 cells were treated with 5-FU (15 µM) for 30 h, and survival was assessed by clonogenic assay, under both normoxic and hypoxic conditions. HCT116 cells were significantly more sensitive to 5-FU treatment when compared to SW837 under normoxic conditions (*p* = 0.016) (Figure 1C). However, this effect was lost under hypoxia, with similar surviving fractions demonstrated in SW837 and HCT116 cells (*p* = 0.398) following 30 h treatment with 5-FU (15 µM) (Figure 1C).

Together, these data demonstrate SW837 cells as an in vitro model of radioresistant rectal cancer under both normoxic and hypoxic conditions and as a chemoresistant model under normoxia.

### 2.2. Characterization of an In Vitro Model of Inherent Radioresistant/Radiosensitive CRC

#### 2.2.1. Basal Proliferation and Cell Cycle Distribution Is Altered in Radioresistant SW837 Cells

**Figure 2:**
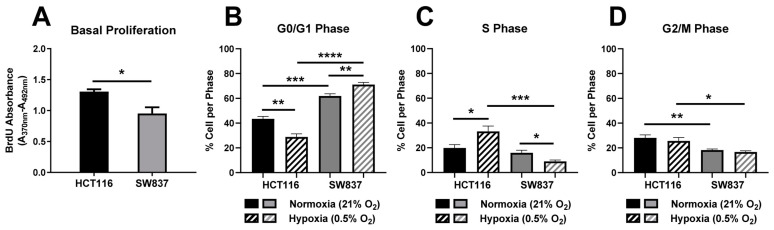
Basal proliferation and cell cycle distribution in HCT116 and SW837 cells. (**A**) Proliferative rates were assessed by BrdU ELISA in HCT116 and SW837 cells. Cell cycle distribution was assessed after 24 h culture under hypoxia (0.5% O_2_) or normoxia (21% O_2_). (**B**) Proportion of HCT116 and SW837 cells in G0/G1 phase under normoxia and hypoxia. (**C**) Proportion of HCT116 and SW837 cells in S phase under normoxia and hypoxia. (**D**) Proportion of HCT116 and SW837 cells in G2/M phase under normoxia and hypoxia. Data are presented as means ± SEMs for four independent experiments. Statistical analysis was performed by paired/unpaired *t*-testing, as appropriate. * *p* < 0.05, ** *p* < 0.01, *** *p* < 0.001, **** *p* < 0.0001.

Having identified SW837 cells as an in vitro model of radioresistant rectal cancer, the potential mechanisms underlying this radioresistance were investigated. Cells with reduced proliferation rates have been demonstrated to be more resistant to cancer therapeutics [15,16]. Basal proliferation rates of HCT116 and SW837 cells were assessed, and SW837 cells were demonstrated to have a significantly decreased basal proliferative capacity when compared to radiosensitive HCT116 cells (*p* = 0.03) (Figure 2A), suggesting that this altered proliferative rate may contribute to the radioresistance of SW837 cells.

The basal cell cycle distribution of cells has also been demonstrated to contribute to radioresistance, with cells in the G2/M phase being the most sensitive to irradiation and cells in the S and G0/G1 phases being more radioresistant [17]. Therefore, the basal cell cycle distribution of HCT116 and SW837 cells under normoxia and hypoxia was assessed by PI staining and flow cytometry to investigate the potential role of altered cell cycle phase distribution in the radioresistance of the SW837 cell line.

Under normoxic conditions, SW837 cells displayed a significantly elevated proportion of cells in the radioresistant G0/G1 phase when compared to radiosensitive HCT116 cells (*p* = 0.0003) (Figure 2B). Under hypoxia, SW837 cells also displayed a significantly elevated proportion of cells in the G0/G1 phase when compared to HCT116 cells (*p* < 0.0001) (Figure 2B). Exposure to hypoxia (24 h) was demonstrated to significantly reduce the proportion of HCT116 cells in the G0/G1 phase (*p* = 0.0028) when compared to normoxic cells (Figure 2B). In contrast, the proportion of SW837 cells in the G0/G1 phase was significantly increased under hypoxic exposure (*p* = 0.0047) when compared to normoxic cells (Figure 2B). Hypoxic exposure induced a significant increase in the proportion of cells in the S phase in HCT116 cells (*p* = 0.02) (Figure 2C) when compared to normoxic conditions. In contrast, hypoxia resulted in a significant decrease in S phase cells in SW837 cells (*p* = 0.033) (Figure 2C) when compared to normoxic conditions.

In addition, under hypoxia, a significant decrease in S phase cells was demonstrated in SW837 cells when compared to HCT116 cells (*p* = 0.0005) (Figure 2C). A significantly smaller proportion of SW837 cells in the radiosensitive G2/M phase was demonstrated when compared to HCT116 cells under normoxia (*p* = 0.005) (Figure 2D). This was also demonstrated under hypoxic conditions, with a significantly lower proportion of cells in the G2/M phase in SW837 cells when compared to HCT116 cells (*p* = 0.011) (Figure 2D).

These data demonstrate a more radioresistant basal proliferative and cell cycle phenotype in SW837 cells when compared to HCT116 cells under both normoxic and hypoxic conditions, which may contribute to the radioresistance of SW837 cells.

#### 2.2.2. Cell Cycle Progression Following Radiation Exposure Is Altered in SW837 and HCT116 Cells under Normoxic and Hypoxic Conditions

Cell cycle progression has also been demonstrated to be associated with radioresponse in cancer. To assess the potential contribution of cell cycle progression in the radioresistance of SW837 cells, cell cycle phase distribution was assessed at 20 min, 6 h, 10 h and 24 h following exposure to a clinically relevant dose of 1.8 Gy X-ray radiation in HCT116 and SW837 cell lines under normoxic and hypoxic conditions by PI staining and flow cytometry.

**Figure 3:**
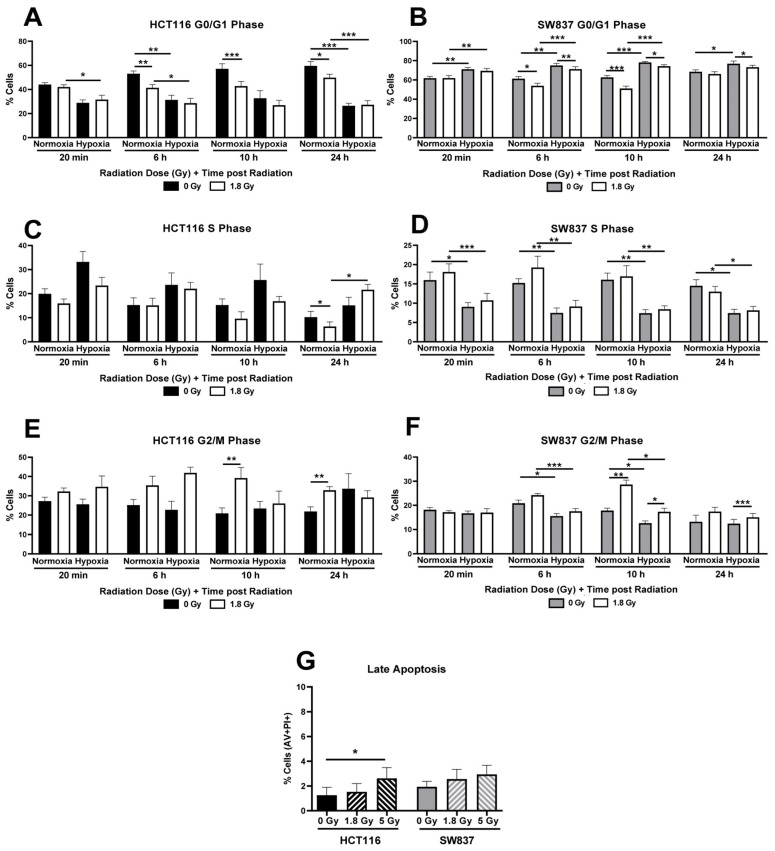
Cell cycle distribution and apoptosis following exposure to radiation in HCT116 and SW837 cells. HCT116 and SW837 cells were placed in normoxia or hypoxia for 24 h prior to being mock-irradiated or exposed to 1.8 Gy X-ray radiation. Cell cycle distribution was assessed at 20 min, 6 h, 10 h and 24 h post-radiation by PI staining and flow cytometry. Proportions of (**A**) HCT116 and (**B**) SW837 cells in G0/G1 phase following radiation, under normoxia or hypoxia. Proportions of (**C**) HCT116 and (**D**) SW837 cells in S phase following radiation, under normoxia or hypoxia. Proportions of (**E**) HCT116 and (**F**) SW837 cells in G2/M phase following radiation, under normoxia or hypoxia. (**G**) The proportions of apoptotic cells among HCT116 and SW837 cells following exposure to 1.8 Gy or 5 Gy radiation were assessed by Annexin V/PI staining and flow cytometry. Data are presented as means ± SEMs for four independent experiments. Statistical analysis was performed by paired ANOVA and post hoc multiple comparison testing. * *p* < 0.05, ** *p* < 0.01, *** *p* < 0.001.

Exposure to a clinically relevant dose of 1.8 Gy radiation induced a significant decrease in the proportion of cells in the G0/G1 phase in HCT116 cells under normoxic conditions and in SW837 cells under normoxic and hypoxic conditions when compared to unirradiated controls (Figure 3A,B).

In addition, radiation exposure induced a decrease in the proportion of HCT116 cells in S phase cells under normoxia when compared to unirradiated controls, whilst no significant alterations were demonstrated in S phase distribution in response to radiation under hypoxic conditions in either HCT116 or SW837 cells (Figure 3C,D). However, G2/M arrest was demonstrated in both HCT116 and SW837 cells following radiation exposure when compared to unirradiated controls (Figure 3E,F). These data demonstrate that cell cycle progression is significantly altered following radiation exposure under normoxic and hypoxic conditions in both radiosensitive HCT116 cells and radioresistant SW837 cells, with differential effects suggesting that cell cycle progression and checkpoint regulation may contribute to the radioresistance of SW837 cells.

The effect of radiation on apoptosis induction in HCT116 and SW837 cells was assessed at 24 h following exposure to clinically relevant doses of 1.8 Gy or 5 Gy X-ray radiation. Similar basal levels of apoptosis were demonstrated in HCT116 and SW837 cell lines. In HCT116 cells, at 24 h post-radiation, a significant increase in the proportion of cells undergoing late apoptosis was demonstrated following 5 Gy radiation when compared to an unirradiated control (*p* = 0.046) (Figure 3G). In contrast, no significant induction of late apoptosis was demonstrated in SW837 cells following either 1.8 Gy or 5 Gy radiation, suggesting that apoptosis is not a major contributor underlying radiation-induced cell death in radioresistant SW837 cells.

#### 2.2.3. Radiation-Induced DNA Damage Is More Efficiently Repaired in Radioresistant SW837 Cells

To investigate whether alterations in DNA damage induction and repair contribute to the differing radiosensitivity of HCT116 and SW837 cancer cells, DNA damage was assessed following irradiation by γH_2_AX-AlexaFluor-488 staining and flow cytometry.

**Figure 4:**
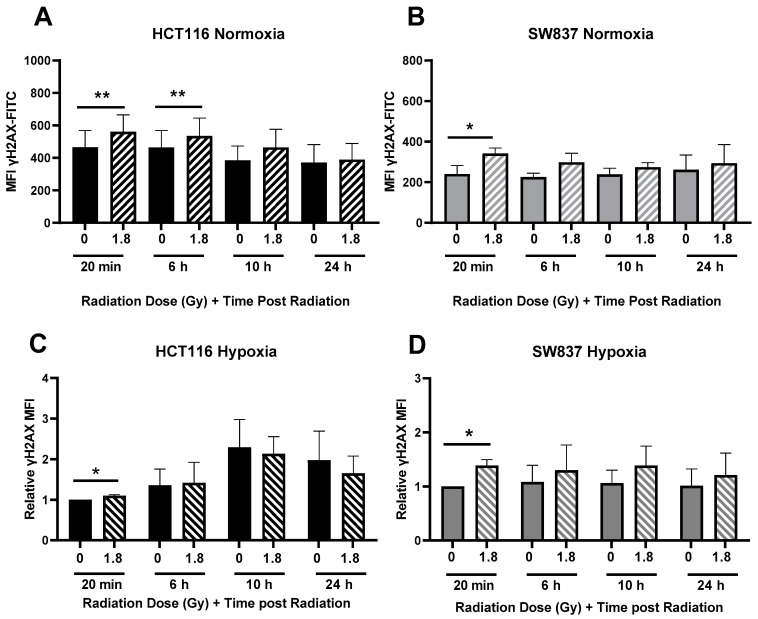
Radiation-induced DNA damage induction and repair in HCT116 and SW837 cells. HCT116 and SW837 cells were irradiated with 1.8 Gy X-ray radiation, and DNA damage was assessed at 20 min, 6 h, 10 h and 24 h post-radiation under normoxia by γH_2_AX fluorescence and flow cytometry. (**A**) DNA damage levels in HCT116 cells following radiation, under normoxic conditions, and (**B**) DNA damage levels in SW837 cells following radiation, under normoxic conditions. HCT116 and SW837 cells were exposed to hypoxia for 24 h prior to irradiation with 1.8 Gy, and DNA damage was assessed at 20 min, 6 h, 10 h and 24 h post-radiation exposure by γH_2_AX fluorescence and flow cytometry. (**C**) DNA damage levels in HCT116 following radiation, under hypoxic conditions. (**D**) DNA damage levels in SW837 cells following radiation, under hypoxic conditions. Data are presented as absolute or relative mean fluorescent intensities (MFIs) ± SEMs for four independent experiments. Statistical analysis was performed by paired *t*-testing. * *p* < 0.05, ** *p*< 0.01.

In both radiosensitive HCT116 and radioresistant SW837 cells, a significant increase in γH_2_AX fluorescence was observed at 20 min post-treatment with a clinically relevant dose of 1.8 Gy radiation when compared to unirradiated controls (Figure 4A,B). In radiosensitive HCT116 cells, radiation-induced DNA damage was still evident at 6 h post-irradiation (Figure 4A). In contrast, in SW837 cells, DNA damage induced by 1.8 Gy was resolved by 6 h post-irradiation (Figure 4B). These data suggest that DNA damage is induced by radiation in both HCT116 and SW837 cells; however, radioresistant SW837 cells demonstrate an enhanced efficiency in the repair of this DNA damage, which may contribute to the radioresistance of these cells.

Having demonstrated enhanced DNA damage repair in radioresistant SW837 cells, under normoxic conditions, DNA damage induction and repair following radiation with a clinically relevant dose of 1.8 Gy was assessed under hypoxic conditions. In radiosensitive HCT116 cells, a significant induction of γH2AX DNA damage was demonstrated at 20 min post-radiation in hypoxic cells (*p* = 0.0224) (Figure 4C). However, by 6 h post-treatment, this significant induction in DNA damage was lost, indicating resolution of radiation-induced damage in HCT116 cells. In hypoxic SW837 cells, a similar induction of DNA damage following radiation was demonstrated at 20 min post-radiation when compared to unirradiated controls (*p* = 0.04) (Figure 4D). γH_2_AX levels had returned to baseline by 6 h post-radiation exposure, suggesting the repair of radiation-induced double-strand breaks (DSBs). These data demonstrate that, under hypoxic conditions, radiation-induced DSBs are repaired by 6 h post-irradiation in both HCT116 and SW837 cells.

### 2.3. Metabolic Reprograming in Radioresistant CRC Cells

#### 2.3.1. SW837 Cells Display a Significantly Altered Basal Transcriptome When Compared to HCT116 Cells

To further characterize HCT116 and SW837 cells and investigate novel mechanisms underlying their differing radiosensitivities, basal transcriptomic profiling was performed.

**Figure 5:**
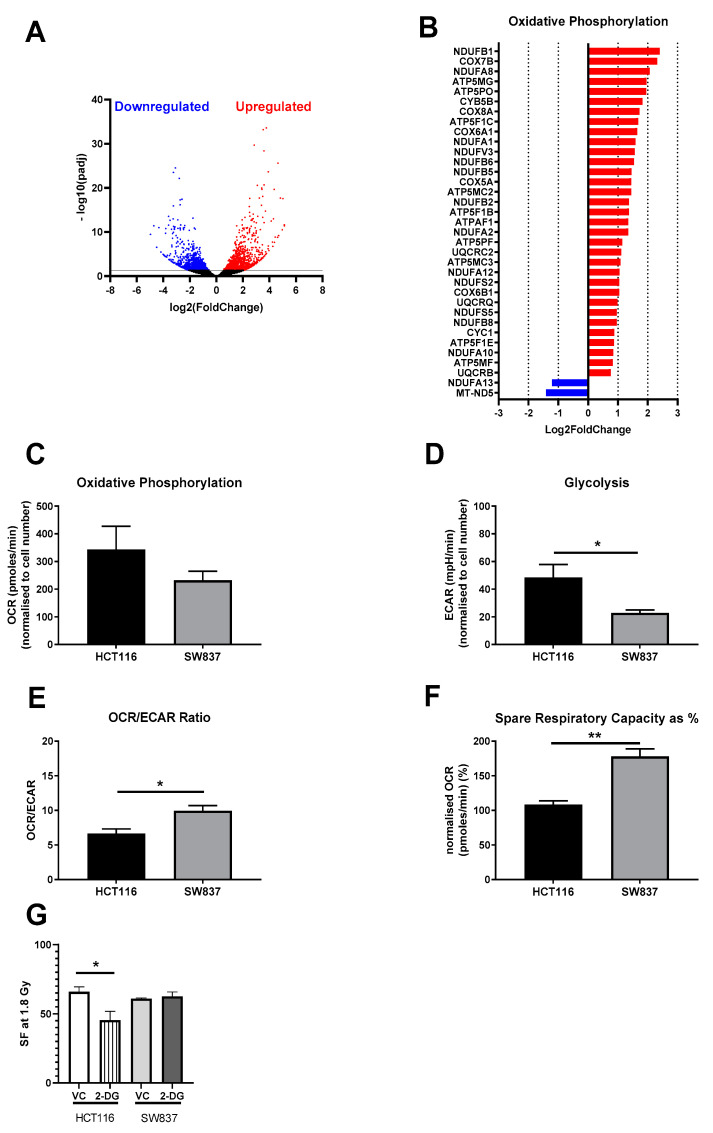
Transcriptomic and metabolic phenotype alterations in radioresistant SW837 cells. (**A**) Volcano plot demonstrating 2461 genes significantly altered in SW837 cells when compared to HCT116 cells. The y-axis shows the −log10 values (*p*-adj), and the x-axis shows the log_2_ values (fold changes). Dots in blue and red represent the significantly downregulated/upregulated genes (2461) in SW837 cells, respectively, when compared to HCT116 cells. Dots in black represent the genes which did not reach statistical significance (*p*-adj > 0.05). (**B**) Differentially expressed genes between HCT116 and SW837 cells were analyzed by IPA software, identifying ‘Oxidative Phosphorylation’ as the most significantly upregulated pathway in SW837 cells when compared to HCT116 cells. Live-cell metabolic profiling by Seahorse technology was assessed in SW837 and HCT116 cells, demonstrating (**C**) oxygen consumption rate, (**D**) extracellular acidification rate, (**E**) OCR: ECAR ratio and (**F**) spare respiratory capacity. (**G**) HCT116 and SW837 cells were treated with 2-DG (10 mM) or a H_2_O vehicle control (VC) for 24 h before irradiation with 1.8 Gy. Radiosensitivity was assessed by clonogenic assay. Data are presented as means ± SEMs for at least three independent experiments. Statistical analysis of differential expression of transcriptomic data was performed using the Wald test, with corrections for multiple comparisons performed using Benjamini–Hochberg correction (FDR). Statistical analysis in metabolic functional analyses and clonogenic assays was performed by *t*-testing. * *p* < 0.05, ** *p* < 0.01.

In total, 24,359 genes were expressed across the two cell lines, with differential expression analysis revealing a total of 2641 genes significantly altered between HCT116 and SW837 cells based on adjusted *p*-values (*p*-adjs) < 0.05 (Figure 5A). A total of 1173 genes were significantly downregulated, and 1468 genes were significantly upregulated in radioresistant SW837 cells when compared to HCT116 cells (Figure 5A), suggesting that alterations in the transcriptome may contribute to the radioresistance of SW837 cells. Having demonstrated differentially altered genes in SW837 cells, the specific functional canonical pathways associated with these alterations were investigated using IPA software (Winter Release 2021), and canonical pathway analysis was performed. Many of the significantly altered canonical pathways identified between HCT116 and SW837 cells are associated with radioresponse, including cell cycle regulation, cellular metabolism, oxidative stress and DNA damage repair (the top 10 most significantly altered canonical pathways are listed in Table 1, with all significantly altered pathways displayed in Supplementary Table S1). Importantly, many of these canonical pathways predicted to be significantly altered were supported by the functional analysis in this study, including alterations to the cell cycle and DNA damage repair.

Interestingly, oxidative phosphorylation was predicted to be the most significantly activated canonical pathway in SW837 cells, with 33 genes (31.1%) of the 106 genes associated with oxidative phosphorylation in the Ingenuity Knowledge Base significantly upregulated and two oxidative phosphorylation genes (1.96%) significantly downregulated in SW837 cells when compared to HCT116 cells (Figure 5B).

#### 2.3.2. Radioresistant SW837 Cells Display Decreased Reliance on Glycolysis 

Having demonstrated that oxidative phosphorylation is the most significantly altered canonical pathway in the transcriptome of radioresistant SW837 cells when compared to radiosensitive HCT116 cells, the live-cell metabolic phenotypes of HCT116 and SW837 cells were assessed by Seahorse profiling. This permits the assessment of two major metabolic pathways: oxidative phosphorylation, represented by oxygen consumption rate (OCR), and glycolysis, represented by extracellular acidification rate (ECAR), in live cells in real time.

Basally, there were no significant alterations in OCR levels between HCT116 and SW837 cell lines (mean OCRs ± SEMs: 343 ± 83.84 versus 232 ± 32.60, respectively) (Figure 5C); however, basal ECAR levels were significantly lower in radioresistant SW837 cells when compared to radiosensitive HCT116 cells (*p* = 0.045) (Figure 5D). Interestingly, radioresistant SW837 cells were demonstrated to have a significantly elevated OCR: ECAR ratio, suggesting a preference for oxidative metabolism when compared to radiosensitive HCT116 cells (*p* = 0.014) (Figure 5E).

To further investigate the metabolic reliance of HCT116 and SW837 cell lines, a series of mitochondrial inhibitors were injected using the Seahorse XFe24 live-cell metabolic assay, and their effects on OCR were measured in real-time. Interestingly, radioresistant SW837 cells were demonstrated to have a significantly higher spare respiratory capacity (SRC) (as a %), which reflects the ability of a cell to adapt and respond to energy demands, when compared to HCT116 cells (*p* = 0.0096) (Figure 5F). Importantly, no significant alterations in mitochondrial mass, reactive oxygen species (ROS) production or mitochondrial membrane potential (MMP) were demonstrated between HCT116 and SW837 cells (Supplementary Figure S1A–C), suggesting that the altered basal metabolic phenotype observed was not simply due to differences in mitochondrial function or mass. Interestingly, treatment of HCT116 cells with the potent glycolysis inhibitor 2-Deoxy-d-glucose (2-DG) significantly (*p* < 0.05) enhanced radiosensitivity at 1.8 Gy when compared to a vehicle control (Figure 5G), an effect not demonstrated in SW837 cells. These data suggest that radioresistant SW837 cells are less reliant on glycolysis and have an enhanced mitochondrial metabolic reserve when compared to radiosensitive HCT116 cells.

### 2.4. The Metabolome of Pre-Treatment Serum Is Significantly Altered in Rectal Cancer Patients Having a Poor Response to neoCRT and Poorer Outcomes

Having demonstrated that a radioresistant phenotype is associated with altered metabolism in rectal cancer in vitro, the potential role of altered energy metabolism in the response of rectal tumors to neoCRT was investigated. The metabolome of pre-treatment serum samples from rectal adenocarcinoma patients (*n* = 52) was assessed by liquid chromatography mass spectrometry (LC-MS) and correlated with subsequent pathological response to neoCRT and other key clinical parameters to investigate the potential role for circulating metabolites as biomarkers associated with response to neoCRT. Patient characteristics are outlined in Table 2.

**Figure 6:**
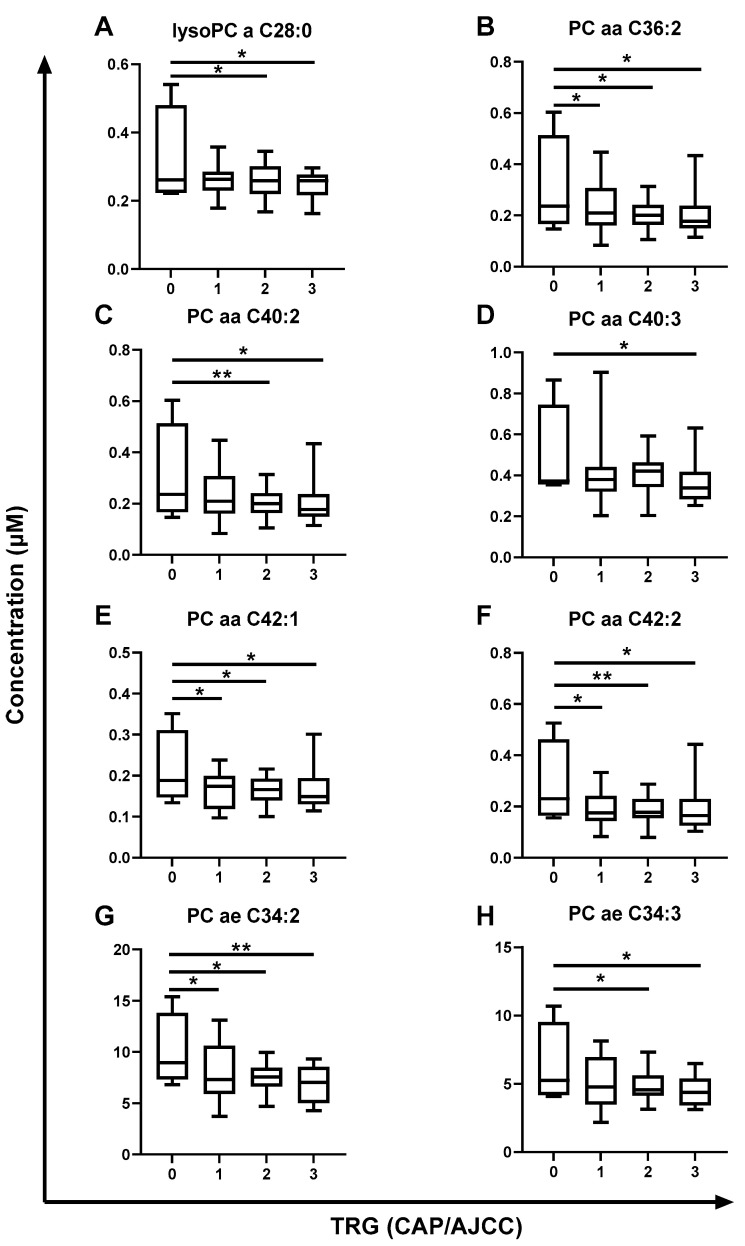
Pre-treatment serum metabolite levels are significantly altered across TRG in rectal cancer patients. Metabolite levels in pre-treatment sera from rectal adenocarcinoma patients (*n* = 52) were assessed by liquid chromatography mass spectrometry (LC-MS) and correlated with subsequent pathological response to neoCRT. (**A**) lysoPC a C28:0, (**B**) PC aa C36:2, (**C**) PC aa C40:2, (**D**) PC aa C40:3, (**E**) PC aa C42:1, (**F**) PC aa C42:2, (**G**) PC ae C34:2 and (**H**) PC ae C34:3 levels were significantly decreased with increasing TRG and worse therapeutic response. TRG 0, *n* = 4; TRG 1, *n* = 14; TRG 2, *n* = 22; TRG 3, *n* = 12. Data are presented as medians ± minima/maxima. Statistical analysis was performed by post hoc unpaired GLM analysis. * *p* < 0.05, ** p < 0.01.

**Figure 7:**
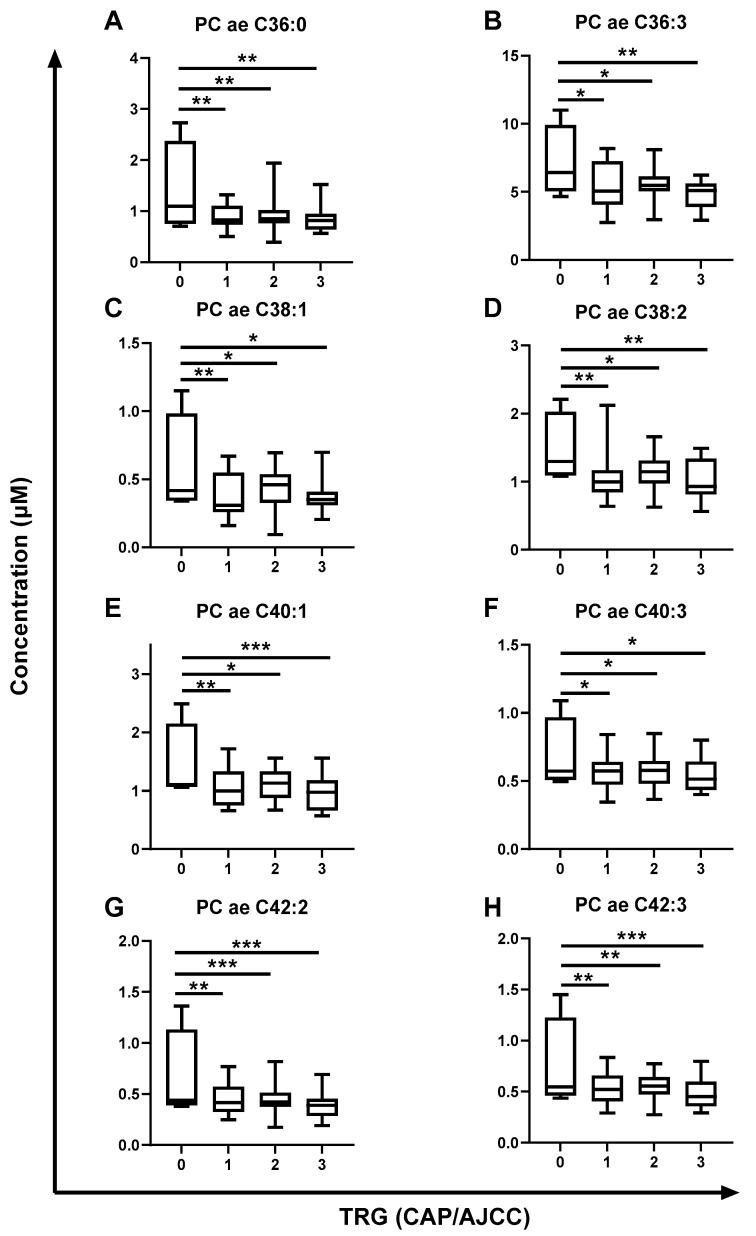
Pre-treatment serum metabolite levels were significantly altered across TRG in rectal cancer patients. Metabolite levels in pre-treatment sera from rectal adenocarcinoma patients (*n* = 52) were assessed by liquid chromatography mass spectrometry (LC-MS) and correlated with subsequent pathological response to neoCRT. (**A**) PC ae C36:0, (**B**) PC ae C36:3, (**C**) PC ae C38:1, (**D**) PC ae C38:2, (**E**) PC ae C40:1, (**F**) PC ae C40:3, (**G**) PC ae C42:2 and (**H**) PC ae C42:2 levels were significantly decreased with increasing TRG and worse therapeutic response. TRG 0, *n* = 4; TRG 1, *n* = 14; TRG 2, *n* = 22; TRG 3, *n* = 12. Data are presented as medians ± minima/maxima. Statistical analysis was performed by post hoc unpaired GLM analysis. * *p* < 0.05, ** p < 0.01, *** *p* < 0.001.

Generalized linear model (GLM) analysis was applied to investigate the association of metabolites with tumor regression grade (TRG) (College of American Pathologists/American Joint Committee of Cancer (CAP/AJCC) Scale), pathological lymph node involvement, differentiation stage and clinical tumor (T) stage, with body mass index (BMI) and sex used as covariates in the analysis. No altered metabolites were demonstrated to be associated with tumor differentiation status or tumor stage. One metabolite (phosphatidylcholine (PC) ae C38:1) was significantly associated with lymph node involvement in pre-treatment sera from rectal cancer patients (Table 3). Interestingly, 16 metabolites were significantly altered depending on TRG (Table 3). Fifteen of the sixteen metabolites significantly associated with therapy response were all PCs. Post hoc multiple comparison GLM analysis demonstrated the significant differences in metabolite concentrations when comparing each TRG (Figure 6 and Figure 7).

Lyso PC a C28:0 was significantly lower in pre-treatment sera from patients with a subsequent poor response (TRG 2 and TRG 3) when compared to those with a complete pathological response (TRG 0) (Figure 6A). Five diacyl (aa) PC metabolites (PC aa C36:2, PC aa C40:2, PC aa C40:3, PC aa C42:1 and PC aa C42:2) were significantly reduced with progressing TRG (Figure 6B–F). Interestingly, the levels of three of these diacyl PCs (PC a C36:2, PC aa C42:1 and PC aa C42:2) were demonstrated to be significantly higher in the sera of patients with a complete response to neoCRT (TRG 0) when compared to each group with a TRG of 1, 2 or 3 (Figure 6B,E,F, respectively).

In addition, the levels of ten acyl alkyl (ae) PCs were significantly reduced in the sera of patients with increasing TRG. Nine of these metabolites were significantly lower in the sera of patients with a TRG of 1, 2 or 3 when compared to those patients with a complete response (TRG 0) (PC ae 34:2, PC ae C36:0, PC ae C36:3, PC ae C38:1, PC ae C38:2, PC ae C40:1, PC ae C40:3, PC ae C42:2 and PC ae C42:3; Figure 6G and Figure 7A–H, respectively).

The relationship between these 16 metabolites and patient outcomes was also investigated using cox regression analysis. Interestingly, decreased levels of 5 of these 16 metabolites in patient sera were significantly associated with poorer recurrence-free survival, and 13 of the 16 metabolites were associated with poorer overall survival (Table 3), further supporting a potential role for these altered metabolites in the pathogenesis of rectal cancer.

Together, these data demonstrate that significant alterations in the levels of 16 metabolites in the pre-treatment sera of rectal cancer patients is associated with subsequent poor pathological response to neoCRT, supporting a potential role for these 16 metabolites as novel circulating predictive markers of treatment response in rectal cancer. 

## 3. Discussion

One of the major clinical challenges in the management of rectal cancer is therapeutic resistance. The mechanisms underpinning radiation resistance in rectal cancer are incompletely understood; thus, this study aimed to identify an in vitro model of inherently radioresistant rectal cancer, to characterize this in vitro model to identify mechanisms underlying radioresistance in rectal cancer and to profile pre-treatment sera from rectal cancer patients to identify alterations that may predict subsequent response to treatment.

The elucidation of the mechanisms underlying radioresistance in rectal cancer has been hampered by a lack of well-characterized pre-clinical model systems, including the low availability of in vitro cell lines of rectal cancer origin. Consequently, most pre-clinical research in the area of rectal cancer is conducted using colon cancer cell lines [9,10]. However, research has demonstrated that not only are right-sided colon cancers and left-sided CRCs anatomically distinct diseases, but also that there are different molecular, immunological and therapeutic implications between these two anatomically distinct sites [12,13,14]. Furthermore, distinct therapeutic strategies are employed in the management of colon and rectal cancer, especially regarding the inclusion of neoadjuvant radiation therapy for locally advanced rectal cancer but not colon cancer [18]. Therefore, the identification and characterization of in vitro radioresistant rectal cancer models is crucial for elucidating the mechanisms underlying treatment resistance.

In this study, SW837 rectal cancer cells were identified as a robust model of inherently radioresistant rectal cancer under both normoxic and hypoxic conditions when compared to the well-characterized radiosensitive HCT116 colon adenocarcinoma cell line (Figure 1). These data support previous findings of SW837 rectal cells as a highly treatment resistant cell line [19,20]. Importantly, hypoxia is a common feature of solid malignancies and a major contributing factor to the development of radioresistance [21,22]. This study demonstrates, for the first time, that SW837 rectal cancer cells remain a robust model of radioresistant rectal cancer under hypoxic conditions (Figure 1B).

The inherent model of radioresistance/radiosensitivity (SW837/HCT116 cells) was subsequently characterized in terms of parameters frequently associated with radioresistance. SW837 cells were demonstrated to have a reduced proliferative rate and display a more radioresistant basal cell cycle distribution when compared to radiosensitive HCT116 cells (Figure 2). SW837 cells have been previously demonstrated to display a higher doubling time than HCT116 cells, reflecting their reduced proliferative rates [23]. Interestingly, elevated proliferation, as reflected by high Ki67 staining, has been previously demonstrated to be associated with favorable prognosis in CRC [24]. The proportion of SW837 cells in the G2/M phase, the most radiosensitive cell cycle phase [17], was significantly lower than that of HCT116 cells. A low proportion of G2/M phase SW837 cells has been previously demonstrated, supporting our findings [19]. In addition, the role of cell cycle regulators in the radioresponse of rectal cancers has been previously demonstrated [8], with reduced expression of p21, an inhibitor of the cell cycle, associated with a radioresistant phenotype in rectal cancer [25]. Hypoxic exposure was demonstrated to affect cell cycle distribution in both HCT116 and SW837 cells (Figure 2 and Figure 3). An enhanced proportion of G0/G1 phase cells and a reduction in S phase and G2/M phase cells was demonstrated in hypoxic SW837 cells when compared to normoxic cells (Figure 2 and Figure 3). These alterations to cell cycle distribution were also demonstrated under hypoxia in an in vitro breast cancer model, which also was more resistant to hypoxia [26]. In contrast, a reduction in G0/G1 and an increase in S phase cells upon hypoxic exposure was demonstrated in HCT116 cells, supporting previous findings in HCT116 cells and potentially contributing to the elevated radioresistance demonstrated in hypoxic HCT116 cells [27,28].

SW837 rectal cancer cells were also demonstrated to display enhanced DNA damage repair when compared to HCT116 cells under normoxic conditions (Figure 4). These data may also suggest impaired DNA damage repair in HCT116 cells, which is supported by the early and sustained high levels of G2/M arrest observed in the cell cycle investigations under normoxia (Figure 3), supporting previous research [29]. Enhanced DNA damage repair capacity is one of the most common features of radioresistant cells, as DSBs are the most critical form of direct radiation-induced damage [30]. In HCT116 cells, under hypoxic conditions, DNA damage induced by 1.8 Gy radiation exposure was resolved by 6 h post-irradiation (Figure 4), concurrent with no significant G2/M arrest following radiation exposure at all time points investigated (Figure 3). These data suggest that hypoxia may aid in the repair of DNA damage, supporting previous studies [28,31,32,33]. Together, these data demonstrate that radioresistant SW837 cells display enhanced DNA damage repair capacity under normoxic conditions, which may be a contributing factor to their radioresistance. In addition, these findings indicate that hypoxic exposure may influence both cell cycle progression and DNA damage repair following radiation to enhance radioresistance.

IPA analysis revealed extensive differences in the basal transcriptome of SW837 and HCT116 cells (Table 1 and Figure 5A). Alterations in cell cycle-related genes were demonstrated, including differential expression of checkpoint-related genes, cyclins and cell cycle regulation, supporting the in vitro data from functional experiments that demonstrated altered basal cell cycle distribution and progression in SW837 cells. Furthermore, DNA damage repair pathway-related genes were demonstrated to be significantly altered between HCT116 and SW837 cells, with enhanced base excision repair and nucleotide excision repair pathway activation in SW837 cells when compared to HCT116 cells, supporting the functional data demonstrating enhanced repair of radiation-induced DNA damage in SW837 cells.

Interestingly, transcriptomic profiling also revealed significant differences in metabolic pathway activation between radioresistant SW837 and radiosensitive HCT116 cells. Oxidative phosphorylation was the most significantly upregulated canonical pathway in SW837 cells, with 30% of oxidative phosphorylation-associated genes demonstrated to be significantly overexpressed in radioresistant SW837 cells when compared to HCT116 cells (Figure 5B). Further interrogation of the metabolic phenotypes of these cells demonstrated significantly lower levels of glycolysis, a decreased reliance on glycolysis (as demonstrated by OCR:ECAR ratios) and a significantly elevated spare respiratory capacity in radioresistant SW837 cells (Figure 5D–F). Interestingly, treatment with the potent glycolysis inhibitor 2-DG significantly sensitized HCT116 cells to 1.8 Gy radiation (Figure 5G), an effect not demonstrated in SW837 cells, further supporting a decreased dependence on glycolysis in these radioresistant cells and suggesting that this is important for the radioresponse of these cells. Importantly, these findings support previous data from our lab, highlighting the importance of oxidative phosphorylation in the radioresistance of esophageal adenocarcinoma [34,35,36] and suggest a common role for metabolic reprogramingin the response of gastrointestinal cancers to radiation. Importantly, cell cycle and DNA repair, which were demonstrated to be altered in radioresistant SW837 cells, require cellular energy availability and are subsequently functionally dependent on energy metabolism, highlighting the potential central role for metabolic pathways in determining tumoral radioresponse in rectal cancer. The importance of mitochondrial metabolism in regulating mechanisms associated with radioresistance has been highlighted previously [37], with evidence demonstrating altered metabolism affecting redox balance, DNA damage repair and the cell cycle in various cancer types. Whilst upregulation of oxidative phosphorylation has been previously demonstrated in CRC [38], this is the first study to demonstrate a decreased reliance on glycolysis in radioresistant rectal cancer and highlights the need for further interrogation of the metabolic profile of radioresistant rectal cancer.

Having demonstrated altered energy metabolism in an in vitro model of inherent radioresistant rectal cancer, the potential role of altered metabolism in the treatment response of rectal cancer patients was investigated. Metabolomic profiling of pre-treatment sera from rectal cancer patients identified 16 metabolites which were significantly associated with subsequent pathological TRG (Figure 6 and Figure 7). Interestingly, 5 and 13 of these metabolites were also significantly associated with poorer recurrence-free and overall survival outcomes, respectively (Table 4), supporting their potential role in the pathogenesis of rectal cancer. These metabolites were primarily PCs, which have been previously demonstrated to be significantly associated with tumorigenesis and therapeutic resistance in many cancer types [39,40]. Recent data from an international consortium demonstrated significant alterations in the plasma levels of metabolites—in particular, PC and sphingomyelin metabolites—associated with CRC stage [41]. One metabolite, PC ae C40:1, identified to be significantly decreased in the plasma of stage IV CRC patients when compared to those with stage I [41], was also significantly reduced in this study, with worsening therapy response.

PCs are involved in the regulation of many metabolic processes and pathways, including those of lipids, lipoproteins and energy metabolism [42]. Supporting this research, a panel of five lipid metabolites, including PC C40:2, was previously identified to be significantly downregulated in the pre-treatment sera of a small cohort of rectal cancer patients [43]. Furthermore, in a study conducted by Jia et al., a panel of 15 metabolic markers, which included three PCs were identified to be predictive of response to neoCRT in the sera of rectal cancer patients, supporting their potential as predictive biomarkers in rectal cancer [44]. Together, these data support the potential utility of PC metabolites as predictive biomarkers of therapeutic response in rectal cancer.

The bioavailability and ease of access of blood samples is a major advantage for the identification of biomarkers. The majority of metabolomic studies profiling biofluids include sera, plasma and urine, and it is widely accepted that the metabolomes of biofluids reflect tumor dynamics [45]. Research utilizing metabolomic approaches for biomarker discovery in cancer has been a growing field of research in recent years. However, while many metabolites have been approved and utilized as clinical biomarkers of disease or response, most of these were identified prior to the technological advancements which have permitted modern metabolomic profiling [46]. Recent advances in metabolomic methods are believed to aid in the progression of metabolomic biomarker studies from research to clinical settings, enhancing the translation of metabolomic biomarkers for clinical utility. While this panel of 16 metabolites associated with response to neoCRT in rectal cancer is promising, this panel will need to be validated in a larger independent cohort of rectal cancer patients and its predictive ability in conjunction with other parameters tested.

This study demonstrates, for the first time, a role for altered energy metabolism, specifically a reduced dependence on glycolysis in the radioresistance of rectal cancer and highlights the potential for metabolites as novel circulating predictive biomarkers of therapeutic response. Further investigation of the role of metabolic reprograming in the radioresistance of rectal cancer may enable the prediction of response to neoCRT and the development of novel treatment strategies targeting mitochondrial metabolism to boost the efficacy of treatment.

## 4. Materials and Methods

### 4.1. Colorectal Adenocarcinoma Cell Lines

The SW837 rectal cancer and the HCT116 colon cancer cell lines were obtained from the European Collection of Cell Cultures (ECACC). The SW837 rectal cancer cell line was originally established from a stage IV rectal adenocarcinoma from a 53-year-old Caucasian male. The HCT116 colon cancer cell line was originally obtained from an adult male colon adenocarcinoma. SW837 cells were maintained in Leibovitz’s (L-15) culture media (Lonza, Basel, Switzerland) supplemented with penicillin-streptomycin (1%) (*v*/*v*) (Lonza), fetal bovine serum (FBS) (10%) (*v*/*v*) (Gibco, Waltham, MA, USA) and L-Glutamine (Lonza) (1%) (*v*/*v*) (complete medium) in non-vented flasks. HCT116 colon cancer cells were maintained in Roswell Park Memorial Institute (RPMI)-1460 medium (Gibco) supplemented with penicillin-streptomycin (1%) (*v*/*v*) and FBS (10%) (*v*/*v*) (complete medium) (complete RPMI (cRPMI)) in vented flasks. Cell lines were cultured at 37 °C in 5% CO_2_/95% humidified air.

### 4.2. X-ray Radiation

All irradiations were performed using an X-Strahl cabinet X-ray irradiator (RS225) (X-Strahl LTD, Walsall, UK) at a dose rate of 1.74 Gy/min. Cells to be irradiated under hypoxic conditions were exposed to X-ray radiation in air-locked containers (Don Whitley Scientific, Bingley, UK).

### 4.3. Clonogenic Assessment of Radiosensitivity under Hypoxia (0.5% O_2_) or Normoxia

HCT116 and SW837 cells in the exponential growth phase were harvested, seeded in cRPMI into 6-well plates at optimized cell densities (HCT116: 0 Gy 500 cell/well, 1.8 Gy 1000 cells/well, 2 Gy 1000 cells/well, 4 Gy 2000 cells/well, 5 Gy 3000 cells/well, 6 Gy 4000 cells/well; SW837: 0 Gy 3000 cells/well, 1.8 Gy 5000 cells/well, 2 Gy 6000 cells/well, 4 Gy 8000 cells/well, 5 Gy 9000 cells/well, 6 Gy 10,000 cells/well) and allowed to adhere to the plate at 37 °C in 5% CO_2_/95% humidified air. Following 6 h incubation, the plates were transferred into a Whitley H35 hypoxystation (Don Whitley Scientific) at 37 °C in 0.5% O_2_/5% CO_2_. Normoxic plates continued to be incubated at 37 °C in 5% CO_2_/95% humidified air (~21% O_2_). Following 24 h incubation under normoxic or hypoxic conditions, the plates remained under either normoxia or hypoxia, and medium was removed from cells to waste and cRPMI was replaced. Cells under hypoxia were treated with deoxygenated RPMI, which had been placed in the hypoxystation 24 h prior to treatment in a vented flask. The following day, cells were exposed to X-ray radiation at required doses or were mock-irradiated. Hypoxic cells were irradiated under hypoxic conditions using air-locked containers. Following 24 h incubation post-irradiation, the cells were removed from hypoxic conditions and the media were removed and replenished with 1.5 mL/well normoxic cRPMI. Cells were incubated at 37 °C in 5% CO_2_/95% humidified air for 7–14 days.

### 4.4. Clonogenic Assessment of 5-FU Sensitivity under Hypoxia (0.5% O_2_) or Normoxia

Cells were harvested by trypsinization, seeded in cRPMI into 6-well plates at optimized densities (HCT116: 500 cells/well, SW837: 5000 cells/well) and allowed to adhere at 37 °C in 5% CO_2_/95% humidified air. At 6 h post-seeding, the cells to be cultured under hypoxic conditions were transferred to a Whitley H35 hypoxystation at 37 °C in 0.5% O_2_/5% CO_2_. Normoxic plates continued to be incubated at 37 °C in 5% CO_2_/95% humidified air (~21% O_2_). After 24 h under hypoxia, medium was removed to waste and cells were treated with 1.5 mL of 5-FU (15 µM) or DMSO vehicle control for 30 h under hypoxic or normoxic conditions, as appropriate. The 5-FU was then removed and replaced with 1.5 mL/well cRPMI. All cells were then incubated at 37 °C in 5% CO_2_/95% humidified air for 7–14 days.

### 4.5. Fixation, Staining and Counting of Clonogenic Assay

Once the colonies had formed but had not yet merged, the medium was removed to waste. For SW837 cells, a volume of 500 µL fixing/staining solution (0.05% (*w*/*v*) crystal violet (Sigma Aldrich, St. Louis, MO, USA) 25% (*v*/*v*) Methanol (Honeywell) in PBS) was added to each well and incubated for 30 min at room temperature (RT°). For HCT116 cells, a volume of 500 µL 4% paraformaldehyde (PFA) (4 °C) (Santa Cruz Biotechnology, Inc., Dallas, TX, USA) was added to the well and incubated for 10 min at RT°. Colonies were then stained with crystal violet solution (0.05% (*w*/*v*)) for 30 min at RT°. The stain was removed to waste, and the wells gently washed with water. The plates were then left to air-dry overnight before being counted.

Colonies were counted using a PC-software-operated colony counter (Gelcount™, Oxford Optronix Ltd., Abingdon, UK, version 1.2.1). Plating efficiencies (PEs), based on control colony counts, were calculated using the following formula: PE = no. colonies/no. cells seeded. The surviving fraction (SF) was calculated using the following formula: SF = No. colonies formed after treatment/(No. cells seeded x PE) [47].

### 4.6. BrdU ELISA

HCT116 and SW837 cells were seeded in triplicate in a 96-well plate (10,000 cells per well) in 100 µL cRPMI and allowed to adhere overnight at 37 °C in 5% CO_2_/95% humidified air. After 24 h, 10 µL BrdU labeling solution was added to each sample, except for appropriate controls. Cell proliferation was assessed at 24 h using the Cell Proliferation BrdU colorimetric ELISA (Roche, Basel, Switzerland), according to the manufacturer’s instructions.

### 4.7. Assessment of Cell Cycle and DNA Damage under Hypoxia (0.5% O_2_) and Normoxia

Cells in the exponential growth phase were seeded into 12-well plates at optimized densities (HCT116: 150,000 cells/well, SW837: 200,000 cells/well) in cRPMI. The cells were allowed to adhere overnight at 37 °C in 5% CO_2_/95% humidified air. Cells for hypoxic culture were transferred to a Whitley H-35 hypoxychamber at 37 °C in 5% CO_2_/0.5% O_2_, while normoxic cells remained at 37 °C in 5% CO_2_/95% humidified air. At 24 h post-hypoxic exposure, medium was removed and replaced with 1 mL complete RPMI per well. Twenty-four hours later, the cells were mock-irradiated or exposed to clinically relevant 1.8 Gy X-ray radiation under normoxic or hypoxic conditions. At 20 min, 6 h, 10 h or 24 h post-irradiation, the cells were collected into 5 mL flow tubes and stained with γH_2_ax-Alexa 488 or PI. Samples were acquired, with a minimum of 10,000 events collected, excluding doublets, using the FACSCanto II flow cytometer (BD Biosciences, Wokingham, UK). γH_2_ax-AlexaFluor-488 was measured on the FITC channel, while PI was measured on the PerCP-Cy5 channel. Data were analyzed using FlowJo™ Version 10.6.2. The gating strategy is outlined in Supplementary Figure S2.

### 4.8. Assessment of Apoptosis by Flow Cytometry

Cells in the exponential growth phase were seeded in cRPMI into 12-well plates at optimized seeding densities (HCT116: 400,000 cells/well, SW837: 500,000 cells/well). Cells were allowed to adhere overnight at 37 °C in 5% CO_2_/95% humidified air. After 24 h, the cells were irradiated with 1.8 Gy or 5 Gy X-ray radiation, while the controls were mock-irradiated. At 24 h post-radiation, supernatants and cells were collected into 5 mL flow tubes. Cells were stained with Annexin-V-FITC for 15 min at 4 °C. Cells were then stained with PI prior to the acquisition of 40,000 cells per tube, excluding doublets, using the FACSCanto II flow cytometer. Annexin-V-FITC was measured on the FITC channel, while PI was measured on the PerCP-Cy5 channel. Data were analyzed using FlowJo™ Version 10.6.2.

### 4.9. Crystal Violet Assay

Medium was removed and SW837 cells were fixed via the addition of 50 µL 1% glutaraldehyde ((*v*/*v*) in PBS) per well for 15 min at RT°. HCT116 cells were fixed with cold 4% PFA (*v*/*v*) for 10 min at RT°. The fixative was removed, and the cells were washed with 50 µL PBS. The cells were stained with 0.1% crystal violet (*w*/*v*) (in dH_2_O) for 30 min at RT°. The cells were gently washed with 50 µL water and allowed to dry overnight at RT°. The crystal violet dye was then eluted via the addition of 50 µL 1% Triton-X100 (in PBS) on an orbital shaker for ~1 h, or until the dye fully eluted, at RT°. The absorbance was read at 595 nm on a VersaMax microplate reader (Molecular Devices, Sunnyvale, CA, USA).

### 4.10. RNA Isolation and Quantification

RNA was isolated from cell lines using the miRNeasy^®®^ Mini Kit (Qiagen, Hilden, Germany), according to the manufacturer’s instructions. RNA was quantified using a Nanodrop 1000 spectrophotometer version 3.1 (Thermo Fisher Scientific, Dublin, Ireland).

### 4.11. Transcriptomic Profiling

Transcriptomic profiling was conducted utilizing mRNA sequencing using the Lexogen QuantSeq 3′ mRNA-Seq. RNA samples were prepared for sequencing using the QuantSeq™ 3′ mRNA-Seq Library prep kit (Lexogen), according to the manufacturer’s instructions, using a starting volume of 50 ng RNA. An equal molar amount of the purified library was pooled for sequencing, with a loading concentration of 320 pM loaded onto the NovaSeq flowcell. Sequencing was performed using the NovaSeq 6000 (Illumina, San Diego, CA, USA) and an SP v1.5 sequencing kit (Illumina) with 1 × 100 bp reads, as per the manufacturer’s instructions.

### 4.12. Transcriptomic Data Analaysis

Raw files were assessed using the BlueBee™ Bioinformatics platform (Lexogen, Vienna, Austria). Raw reads were trimmed and aligned for automated gene counting. Once gene reads and counts were complete, differential expression analysis was performed using the DESeq2 R script extension within BlueBee software (https://www.lexogen.com accessed October 2021).

### 4.13. IPA Analysis

Significantly differentially expressed genes and corresponding log_2_ fold change values were imported into IPA bioinformatics software (Qiagen, Redwood City, CA, USA, Winter Release 2021). Core analysis in IPA was performed, which utilizes the Qiagen Knowledge Base, to identify networks and predict specific biological function and pathway involvement in the uploaded experimental transcriptomic dataset.

Canonical pathway analysis in IPA, utilizing the Qiagen Knowledge Base, was utilized to predict the involvement and activation or inhibition of specific biological pathways in the experimental dataset. *p*-values denoted the significance between the overlap of inputted experimental data and the Ingenuity Knowledge Base, indicating confidence in pathway involvement. Z-scores refer to software prediction of the activation or inhibition of each affected canonical pathway, with a Z-score ≥ 2 or ≤−2 indicating significant activation or inhibition of each pathway, respectively.

### 4.14. Live-Cell Metabolic Profiling by Seahorse XFtechnology

Cells were seeded at optimized seeding densities (HCT116: 10,000 cells/well, SW837: 30,000 cells/well) in a 24-well cell culture XFe24 microplate (Agilent Technologies, Santa Clara, CA, USA) at a volume of 100 µL of complete RPMI and allowed to adhere at 37 °C in 5% CO_2_/95% humidified air. At 5 h post-seeding, an additional 150 µL complete medium was added to each well. Following 24 h, the treatment was removed, and cells were washed with unbuffered Seahorse XF Base DMEM (Agilent Technologies, Santa Clara, CA, USA) (supplemented with 10 mM glucose (Sigma), 10 mM sodium pyruvate (Sigma) and L-glutamine) and placed in a non-CO_2_ incubator for 1 h at 37 °C. Oxygen consumption rate (OCR) and extracellular acidification rate (ECAR) were measured using the Seahorse XFe24 XF Analyzer, according to the manufacturer’s instructions. Briefly, the Seahorse XF analyzer measures OCR and ECAR in live cells by measuring the concentrations of dissolved oxygen and free protons using solid-state sensor probes residing 200 microns above the cell monolayer. The instrument measures for 2–5 min then calculates the OCR and ECAR. Three baseline measurements of OCR and ECAR were taken over 24 min, consisting of 2 repetitions of mix (3 min)/wait (2 min)/measurement (3 min), to establish basal respiration. These steps were repeated following the injection of a series of mitochondrial targeting compounds (oligomycin A (2 µg/mL, Sigma), carbonyl cyanide 4-(trifluoromethoxy) phenylhydrazone (FCCP) (5 µM, Sigma) and antimycin-A (2 µM, Sigma)), as described previously [36]. The injection of these compounds permitted the further investigation of the metabolic phenotypes of the cells being tested. OCR:ECAR ratios were calculated by dividing the basal OCR values by the basal ECAR values of each cell line [48]. All OCR/ECAR readings were normalized to cell number using the crystal violet assay (described in detail in Section 4.10). Briefly, following metabolic profiling, the cells were fixed and stained with crystal violet. The crystal violet was subsequently eluted from the cells, and absorbance was measured using a VersaMax microplate reader. OCR and ECAR readings were normalized to cell number using relative crystal violet absorbance values.

### 4.15. 2-DG Clonogenic Assay

HCT116 and SW837 cells were seeded in cRPMI into 6-well plates (HCT116: 0 Gy 500 cell/well, 1.8 Gy 1000 cells/well; SW837: 0 Gy 3000 cells/well, 1.8 Gy 5000 cells/well) and allowed to adhere at 37 °C in 5% CO_2_/95% humidified air. At 24 h post-seeding, cells were treated with 2-DG (10 mM) or H_2_O vehicle control in cRPMI for 24 h and then irradiated with 1.8 Gy X-ray radiation, or mock-irradiated, and placed at 37 °C in 5% CO_2_/95% humidified air for 7–14 days. Colonies were fixed and counted, as previously described.

### 4.16. Patient Ethics, Treatment and Sample Collection

Following ethical approval (Regional Committee for Medical and Health Research Ethics of South-East Norway (reference number: REK 2013/152) and the Institutional Review Board (reference number: 12-106)) and written informed consent, serum samples were taken from rectal adenocarcinoma patients between October 2013 and November 2017 at Akershus University Hospital (Lørenskog, Norway) (ClinicalTrials.gov NCT01816607) prior to neadjuvant chemoradiation therapy.

Eligible patients were 18 years or older with no prior radiation therapy for pelvic neoplasia. Patients had histologically verified rectal adenocarcinoma that was considered high-risk by magnetic resonance imaging: T2 cases that presented a tumor threatening the anal sphincter muscles, T3 cases that had a mesorectal fascia margin of less than 2 mm, T4 cases (organ-infiltrating tumor) or cases that involved pelvic cavity lymph nodes (N1-2 disease). The patients were treated according to the prevailing national guidelines with neoCRT and radical pelvic surgery. The absence of metastatic disease at the time of diagnosis was established based on computed tomography scans of the thoracic and abdominal cavities. Chemotherapy consisted of capecitabine, FLOX (fluorouracil, leucovorin and oxaliplatin) or FLV (5-FU and leucovorin). Radiation therapy was delivered in either 25 fractions of 2 Gy or 5 fractions of 5 Gy. The resected tumor specimens were histologically evaluated by the specialist in gastrointestinal pathology for local treatment response (ypTN stage) and tumor regression grade. According to this system, grade 0 represents complete absence of tumor cells, grade 1 represents <5% residual tumor cells (near-complete or complete response), grade 2 represents 5–50% residual tumor cells and grade 3 represents >50% residual tumor cells [49]. All patients were followed systematically with clinical examination and CT scans at 3, 6, 12, 18 and 24 months and then every year for a total of 5 years after surgery. Overall survival was verified using the Norwegian population registry, and at the last censoring date on 15 June 2022, median follow-up for the 52 patients was 63 months, the minimum was 9 months and the maximum was 103 months. At this time, 18 patients were deceased.

### 4.17. Preparation of Patient Samples

For serum preparation, whole blood was drawn in plain tubes with no additives for centrifugation to separate the serum, which was left on ice for no more than 1 h before storage at −80 °C.

### 4.18. Metabolomic Profiling by AbsoluteIDQ^®®^ p180 Assay

Serum samples were thawed and centrifuged at 2750× *g* for 5 min at 4 °C prior to metabolomic analysis. Metabolites were identified and quantified using the AbsoluteIDQ^®®^ p180 assay (Biocrates Life Sciences, Innsbruck, Austria), according to the manufacturers’ instructions. Detailed sample preparation and analysis were previously described [50]. Briefly, 10 µL quantities of serum were added to the 96-well plate and dried under a stream of nitrogen. A total of 50 µL of 5% phenyl isothiocyanate solution was added and incubated for 25 min atRT°. Following incubation, the plate was dried for 60 min under the nitrogen stream. The extraction solvent (5 mM ammonium acetate in methanol, 300 µL) was added to each well, and the plate was subsequently shaken for 30 min. The plate was centrifugated at 500× *g* for 2 min to obtain the eluate, and 150 µL of eluate was diluted with 150 µL of HPLC grade water for the liquid chromatography-tandem mass spectrometry (LC-MS/MS) run. A total of 50 µL of eluate was diluted with 450 µL mobile phase for the flow injection analysis-tandem mass spectrometry (FIA-MS/MS) run.

The data were acquired on a SCIEX QTRAP 6500plus mass spectrometer coupled to SCIEX ExionLC™ Series UHPLC capability. During the LC-MS/MS run, a UHPLC column provided with an AbsoluteIDQ^®®^ p180 kit was installed for metabolite separation, and water and acetonitrile (both with 0.2% formic acid added) were used as mobile phase A and B, respectively. Amino acids (*n* = 21) and biogenic amines (*n* = 21) were identified and quantified in positive mode. For the FIA-MS/MS analyses, methanol was employed as the running solvent, and 40 acylcarnitines, 14 lysophosphatidylcholines (lysoPC), 38 acyl/acyl phosphatidylcholines (PC aa), 38 acyl/alkyl phosphatidylcholines (PC ae), 15 sphingomyelins (SMs) and the sum of hexoses (H1) were identified and quantified in positive mode. In this assay, all metabolites were quantified by the multiple reaction monitoring (MRM) method, which was optimized and provided by Biocrates Life Sciences. Data acquisition was conducted using AB Sciex Analyst^®®^ software, version 1.7.2.

### 4.19. Data Processing and Metabolite Quantification

Amino acids and biogenic amines were quantified based on isotopically labeled internal standards and 7-point calibration curves using AB Sciex Analyst^®®^ version 1.7.2 software. Other metabolites, such as acylcarnitines, lysoPCs, PCs, SMs and hexoses, were semi-quantified using 14 internal standards in MetIDQ™ software version 8.7.1(Biocrates Life Sciences). Data quality was evaluated by checking the accuracy and reproducibility of QC samples included in the p180 kit. Finally, the concentrations of metabolites were reported in µM. For further statistical analyses, metabolites were included only when the concentrations of metabolites were above the limit of detection (LOD) in more than 50% of samples.

### 4.20. Statistical Analysis

All statistical analysis and graphing were performed using Graphpad Prism v9 software or SPSS for Mac v28. Data are presented as means ± standard errors of the means (SEMs) or as hazard ratios (HRs) with 95% confidence intervals (95% CIs) for survival analysis throughout. Statistical comparisons were carried out using analysis of variance (ANOVA) testing, post hoc Tukey’s multiple comparisons testing or *t*-testing, depending on the experimental set up, as described in the figure legends. Metabolomic data analysis was performed by unpaired *t*-testing, Cox regression testing, GLM analysis or as described in the figure legends. For transcriptomic data analysis, BlueBee and IPA software were utilized for statistical analysis. BlueBee differential expression analysis utilized Wald testing with Benjamini–Hochberg correction, while IPA utilized Fisher’s exact test, as stated in the figure/table legends. Genes were considered differentially expressed with an adjusted *p*-value (*p*-adj) < 0.05.

## Figures and Tables

**Table 1:** Top 10 canonical pathways significantly altered in SW837 cells when compared to HCT116 cells.

Ingenuity Canonical Pathways	*p*-Value	z-Score
Oxidative Phosphorylation	1.95 × 10^−9^	5.145
Sirtuin Signalling Pathway	6.31 × 10^−9^	−2.496
EIF2 Signalling	3.80 × 10^−8^	2.746
Mitochondrial Dysfunction	9.12 × 10^−8^	NaN
Coronavirus Pathogenesis Pathway	3.98 × 10^−6^	−2.287
Regulation of eIF4 and p70S6K Signalling	8.71 × 10^−6^	−0.707
Axonal Guidance Signalling	1.02 × 10^−5^	NaN
Actin Cytoskeleton Signalling	1.15 × 10^−5^	−0.632
BAG2 Signalling Pathway	1.91 × 10^−5^	−0.632
mTOR Signalling	3.39 × 10^−5^	0.894

IPA canonical pathway analysis was utilized to predict pathway activation based on input transcriptomic data and the Ingenuity Knowledge Base. The *p*-values denote the significance levels between the overlap of the inputted experimental data and the knowledge base. Z-scores indicate the significance of the activation/inhibition status of each predicted pathway. Statistical analysis was performed using Fisher’s exact testing.

**Table 2:** Patient characteristics of rectal cancer patients used in metabolomic analysis of pre-treatment serum samples.

		(*n* = 52)
Gender	Male (*n*)	35
	Female (*n*)	17
Age	Mean (y)	62
	Range (y)	41–79
Histology	Adenocarcinoma (*n*)	52
Clinical T Stage	2 (*n*)	3
	3 (*n*)	25
	4 (*n*)	24
Pathological Nodal Involvement	Yes (*n*)	25
	No (*n*)	27
Treatment	NeoCRT (*n*)	52
TRG (CAP/AJCC Scale)	0 (*n*)	4
	1 (*n*)	14
	2 (*n*)	22
	3 (*n*)	12
Recurrence-Free Survival	Median	63
	Minimum	2
	Maximum	103
Overall Survival (Months)	Median	63
	Minimum	9
	Maximum	103

Abbreviations: y, years; T stage, tumor stage; neoCRT, neoadjuvant chemoradiation therapy; TRG, tumor regression grade.

**Table 3:** GLM analysis of metabolite alterations in pre-treatment sera from rectal cancer patients significantly associated with clinical parameters.

Metabolite	TRG (CAP/AJCC Scale) *p*-Value (FDR-Corrected)	Lymph Node Positivity *p*-Value (FDR-Corrected)
LysoPC a C28:0	0.0143	
PC aa C36:2	0.0322	
PC aa C40:2	0.0143	
PC aa C40:3	0.025	
PC aa C42:1	0.0322	
PC aa C42:2	0.0243	
PC ae C34:2	0.0184	
PC ae C34:3	0.0250	
PC ae C36:0	0.0072	
PC ae C36:3	0.0322	
PC ae C38:1	0.0207	0.0473
PC ae C38:2	0.0207	
PC ae C40:1	0.0033	
PC ae C40:3	0.0322	
PC ae C42:2	0.0010	
PC ae C42:3	0.0021	

GLM analysis was used to estimate the significantly different features based on TRG (CAP/AJCC), with sex and BMI used as covariates. Based on metabolite levels from pre-treatment sera of *n* = 52 rectal adenocarcinoma patients. TRG 0, *n* = 4; TRG 1, *n* = 14; TRG 2, *n* = 22; TRG, 3 *n* = 12. *p*-values (FDR-corrected) are corrected for multiple comparisons using the Benjamini–Hochberg (BH) procedure.

**Table 4:** Decreased pre-treatment serum levels of metabolites were significantly associated with recurrence-free (*n* = 41) and overall survival (*n* = 52), respectively, in rectal cancer patients.

	Recurence-Free Survival		Overall Survival	
Metabolite	HR (95% CI)	*p*-Value	HR (95% CI)	*p*-Value
LysoPC a C28:0	0.145 (0.000–6103.4)	0.722	0.062 (0.000–316–7)	0.523
PC aa C36:2	0.984 (0.966–1.001)	0.070	0.992 (0.981–1.004)	0.202
PC aa C40:2	0.003 (0.000–161.4)	0.300	0.000 (0.000–0.177)	0.017
PC aa C40:3	0.042 (0.000–38.58)	0.363	0.008 (0.000–1.034)	0.052
PC aa C42:1	0.000 (0.000–1.702)	0.057	0.000 (0.000–0.039)	0.014
PC aa C42:2	0.000 (0.000–0.009)	0.012	0.000 (0.000–0.017)	0.005
PC ae C34:2	0.808 (0.558–1.170)	0.258	0.705 (0.542–0.917)	0.009
PC ae C34:3	0.717 (0.403–1.275)	0.258	0.645 (0.432–0.963)	0.032
PC ae C36:0	0.042 (0.002–0.986)	0.042	0.088 (0.009–0.872)	0.038
PC ae C36:3	0.735 (0.446–1.212)	0.228	0.688 (0.480–0.985)	0.041
PC ae C38:1	0.022 (0.000–3.152)	0.132	0.020 (0.000–0.826)	0.039
PC ae C38:2	0.042 (0.002–0.821)	0.037	0.087 (0.014–0.540)	0.009
PC ae C40:1	0.132 (0.009–1.894)	0.136	0.101 (0.014–0.754)	0.025
PC ae C40:3	0.006 (0.000–3.848)	0.120	0.003 (0.000–0.207)	0.008
PC ae C42:2	0.008 (0.000–0.077)	0.008	0.001 (0.000-0.067)	0.002
PC ae C42:3	0.000 (0.000–0.155)	0.011	0.001 (0.000-0.064)	<0.001

Adjusted Cox regression analysis was used to estimate associations between the 16 selected metabolites and recurrence-free and overall survival of the 52 patients. Of note, 11 patients were missing recurrence-free survival data, leaving 41 patients for this analysis. Data are presented as hazard ratios (HRs) and 95% confidence intervals (95% CIs). At the last censoring date, 15 June 2022, median follow-up was 63 months, the minimum was 9 months, and the maximum was 103 months. A total of 18 of the 52 patients were deceased.

## Data Availability

The data presented in this study are available upon reasonable request from the corresponding author. The data are not publicly available as there are restrictions on data processing in line with participant consent.

## Supplementary Materials

The supporting information can be downloaded at: https://www.mdpi.com/article/10.3390/ijms24087082/s1.
