# Supplementary material for: Energy Metabolism Is Altered in Radioresistant Rectal Cancer
Source: Int J Mol Sci. 2023 Apr 11;24(8):7082. doi: 10.3390/ijms24087082 (PMC10138551; doi:10.3390/ijms24087082)
Supplement: Supplementary file 1 [file ijms-24-07082-s001.zip › ijms-2184156-supplementary.pdf]

Supplementary Data

Supplementary Table S1: Canonical pathways significantly altered in SW837 cells, when compared to HCT116 cells

Ingenuity Canonical Pathways altered in SW837 cells	-log(p-value)
Oxidative Phosphorylation	8.71E+00
Sirtuin Signaling Pathway	8.20E+00
EIF2 Signaling	7.42E+00
Mitochondrial Dysfunction	7.04E+00
Coronavirus Pathogenesis Pathway	5.40E+00
Regulation of eIF4 and p70S6K Signaling	5.06E+00
Axonal Guidance Signaling	4.99E+00
Actin Cytoskeleton Signaling	4.94E+00
BAG2 Signaling Pathway	4.72E+00
mTOR Signaling	4.47E+00
Signaling by Rho Family GTPases	4.36E+00
FAT10 Signaling Pathway	4.26E+00
Semaphorin Neuronal Repulsive Signaling Pathway	4.25E+00
p53 Signaling	4.21E+00
Regulation of Actin-based Motility by Rho	4.18E+00
Cell Cycle: G2/M DNA Damage Checkpoint Regulation	4.14E+00
RHOGDI Signaling	4.06E+00
Pyrimidine Deoxyribonucleotides De Novo Biosynthesis I	4.04E+00
Cell Cycle: G1/S Checkpoint Regulation	4.00E+00
Role of p14/p19ARF in Tumor Suppression	3.91E+00
Tight Junction Signaling	3.86E+00
Protein Ubiquitination Pathway	3.85E+00
Molecular Mechanisms of Cancer	3.84E+00
Clathrin-mediated Endocytosis Signaling	3.75E+00
Semaphorin Signaling in Neurons	3.73E+00
Cholesterol Biosynthesis I	3.68E+00
Cholesterol Biosynthesis II (via 24,25-dihydrolanosterol)	3.68E+00
Cholesterol Biosynthesis III (via Desmosterol)	3.68E+00
ILK Signaling	3.61E+00
Sertoli Cell-Sertoli Cell Junction Signaling	3.59E+00
Sumoylation Pathway	3.55E+00
Inhibition of ARE-Mediated mRNA Degradation Pathway	3.45E+00
Germ Cell-Sertoli Cell Junction Signaling	3.42E+00
Aryl Hydrocarbon Receptor Signaling	3.40E+00
3-phosphoinositide Biosynthesis	3.38E+00
Virus Entry via Endocytic Pathways	3.35E+00
D-myo-inositol (1,4,5,6)-Tetrakisphosphate Biosynthesis	3.35E+00
D-myo-inositol (3,4,5,6)-tetrakisphosphate Biosynthesis	3.35E+00
Glioblastoma Multiforme Signaling	3.32E+00
ERK/MAPK Signaling	3.31E+00
Glycogen Degradation II	3.25E+00
Estrogen Receptor Signaling	3.22E+00

Superpathway of Cholesterol Biosynthesis	3.21E+00
Integrin Signaling	3.16E+00
3-phosphoinositide Degradation	3.10E+00
Colorectal Cancer Metastasis Signaling	3.01E+00
Actin Nucleation by ARP-WASP Complex	3.01E+00
D-myo-inositol-5-phosphate Metabolism	2.93E+00
MSP-RON Signaling In Cancer Cells Pathway	2.81E+00
Kinetochore Metaphase Signaling Pathway	2.79E+00
Glycogen Degradation III	2.77E+00
RHOA Signaling	2.68E+00
WNT/Î²-catenin Signaling	2.62E+00
CSDE1 Signaling Pathway	2.60E+00
Protein Kinase A Signaling	2.59E+00
Pancreatic Adenocarcinoma Signaling	2.58E+00
Dolichyl-diphosphooligosaccharide Biosynthesis	2.56E+00
Role of BRCA1 in DNA Damage Response	2.53E+00
Hereditary Breast Cancer Signaling	2.50E+00
Ephrin Receptor Signaling	2.49E+00
RAR Activation	2.46E+00
Ferroptosis Signaling Pathway	2.43E+00
Leukocyte Extravasation Signaling	2.42E+00
Cyclins and Cell Cycle Regulation	2.35E+00
Pulmonary Fibrosis Idiopathic Signaling Pathway	2.33E+00
Huntington's Disease Signaling	2.32E+00
Superpathway of Inositol Phosphate Compounds	2.30E+00
TR/RXR Activation	2.29E+00
CXCR4 Signaling	2.23E+00
BEX2 Signaling Pathway	2.16E+00
Cleavage and Polyadenylation of Pre-mRNA	2.14E+00
Senescence Pathway	2.08E+00
ID1 Signaling Pathway	2.07E+00
Glucocorticoid Receptor Signaling	2.06E+00
Pyrimidine Ribonucleotides Interconversion	2.05E+00
Mitotic Roles of Polo-Like Kinase	2.04E+00
RAC Signaling	2.00E+00
HER-2 Signaling in Breast Cancer	2.00E+00
HGF Signaling	1.97E+00
Production of Nitric Oxide and Reactive Oxygen Species in Macrophages	1.95E+00
Hepatic Fibrosis Signaling Pathway	1.94E+00
Protein Citrullination	1.92E+00
Myo-inositol Biosynthesis	1.92E+00
Epoxysqualene Biosynthesis	1.90E+00
S-methyl-5'-thioadenosine Degradation II	1.90E+00
Leucine Degradation I	1.90E+00
Ephrin B Signaling	1.89E+00
Netrin Signaling	1.89E+00
Iron homeostasis signaling pathway	1.89E+00
Pyrimidine Ribonucleotides De Novo Biosynthesis	1.88E+00

Cardiac Î²-adrenergic Signaling	1.88E+00
Remodeling of Epithelial Adherens Junctions	1.86E+00
Chronic Myeloid Leukemia Signaling	1.86E+00
Paxillin Signaling	1.86E+00
Epithelial Adherens Junction Signaling	1.86E+00
CDK5 Signaling	1.85E+00
PCP (Planar Cell Polarity) Pathway	1.84E+00
Human Embryonic Stem Cell Pluripotency	1.83E+00
Chondroitin Sulfate Degradation (Metazoa)	1.82E+00
Ovarian Cancer Signaling	1.82E+00
Phagosome Maturation	1.82E+00
Coronavirus Replication Pathway	1.80E+00
Small Cell Lung Cancer Signaling	1.79E+00
Hypoxia Signaling in the Cardiovascular System	1.78E+00
Relaxin Signaling	1.75E+00
Caveolar-mediated Endocytosis Signaling	1.73E+00
TNFR2 Signaling	1.73E+00
Cell Cycle Regulation by BTG Family Proteins	1.72E+00
HOTAIR Regulatory Pathway	1.72E+00
Cell Cycle Control of Chromosomal Replication	1.70E+00
Dermatan Sulfate Degradation (Metazoa)	1.69E+00
Macropinocytosis Signaling	1.68E+00
Endocannabinoid Cancer Inhibition Pathway	1.67E+00
Selenocysteine Biosynthesis II (Archaea and Eukaryotes)	1.66E+00
IGF-1 Signaling	1.65E+00
HIPPO signaling	1.62E+00
Gap Junction Signaling	1.55E+00
Apelin Adipocyte Signaling Pathway	1.53E+00
Glioma Signaling	1.51E+00
Role of Osteoblasts, Osteoclasts and Chondrocytes in Rheumatoid Arthritis	1.49E+00
fMLP Signaling in Neutrophils	1.48E+00
NAD Signaling Pathway	1.47E+00
RAN Signaling	1.46E+00
Putrescine Degradation III	1.46E+00
NER (Nucleotide Excision Repair, Enhanced Pathway)	1.46E+00
Guanine and Guanosine Salvage I	1.45E+00
Adenine and Adenosine Salvage I	1.45E+00
UDP-N-acetyl-D-galactosamine Biosynthesis II	1.42E+00
IL-1 Signaling	1.41E+00
EGF Signaling	1.39E+00
April Mediated Signaling	1.39E+00
Glutathione Redox Reactions I	1.34E+00
ATM Signaling	1.34E+00
B Cell Activating Factor Signaling	1.33E+00
TNFR1 Signaling	1.31E+00
Guanosine Nucleotides Degradation III	1.30E+00

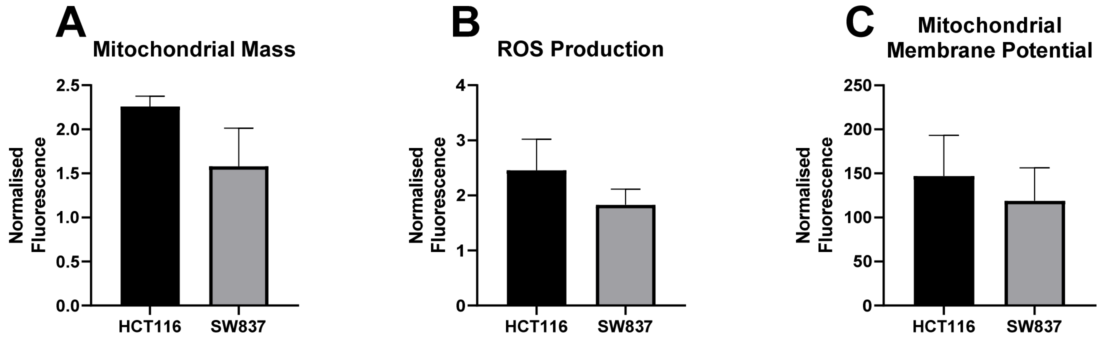

Supplementary Figure S1: Mitochondrial function was assessed in HCT116 and SW837 cells using fluorescent probes. **A)** Mitochondrial mass was assessed using MitoTracker Green FM. **B)** ROS production was assessed using 2,7 DCF-DA. **C)** Mitochondrial membrane potential was assessed using Rhodamine-123. Data is presented as mean ± SEM for 3 independent experiments. Statistical analysis was performed by unpaired *t*-testing.

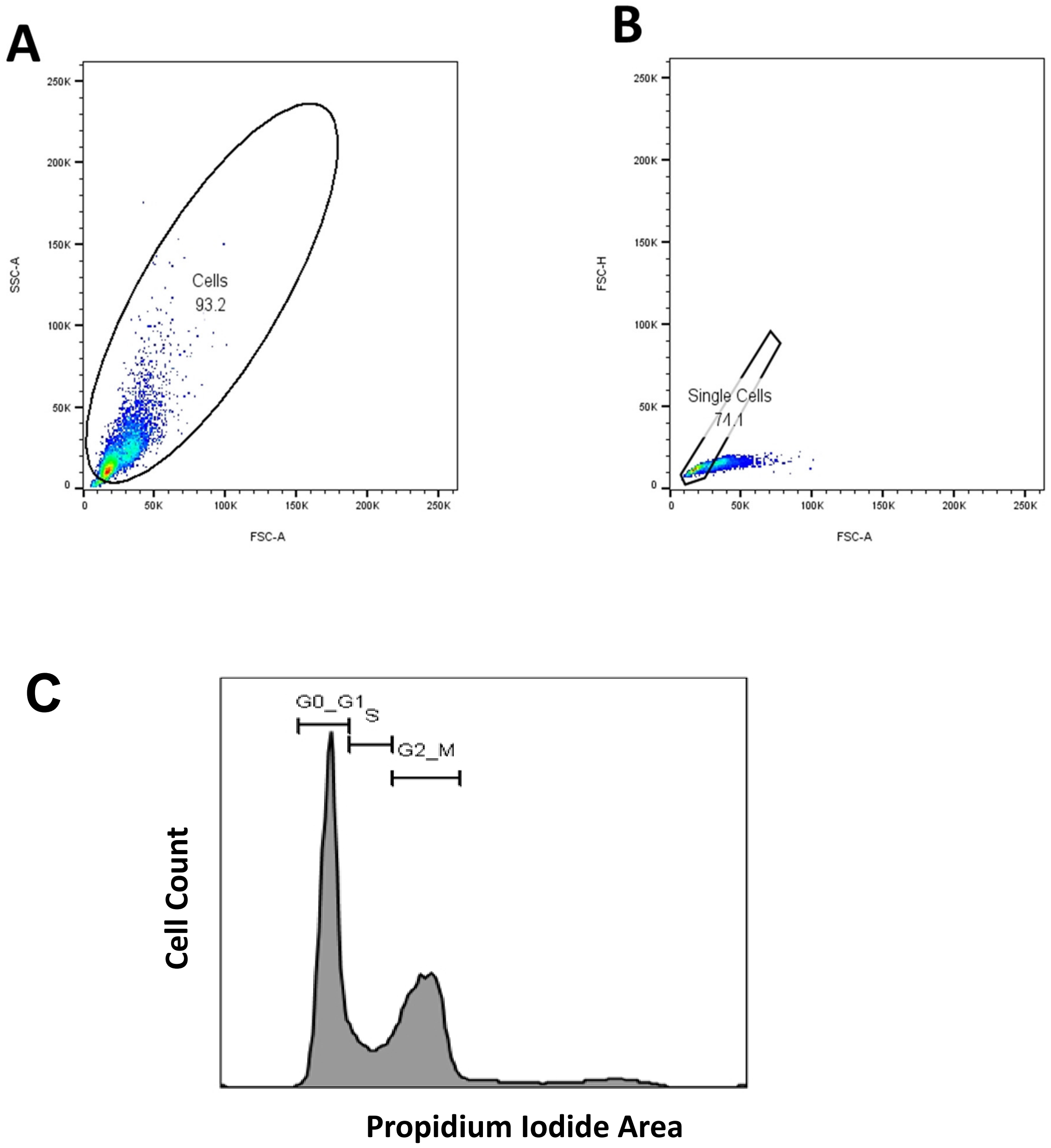

Supplementary Figure S2: Representative images of gating strategy employed for cell cycle flow cytometry experiments. **A)** Gating on cells to exclude debris. **B)** Gating on single cells, to exclude doublets. **C)** Representative histogram plot of cell cycle phase markers.

Supplementary Methods

S.1 Mitochondrial function

Three surrogate markers of mitochondrial function, reactive oxygen species (ROS), mitochondrial membrane potential and mitochondrial mass were assessed using a series of fluorescent probes (2,7 dichlorofluorescein (DCF)(5 μM), Rhodamine-123 (5 μM) and MitoTracker Green^{FM} (0.3 μM). HCT116 cells (10,000 cells per well) and SW837 cells (30,000 cells per well) were seeded in triplicate in a 96-well plate, and allowed to adhere overnight in 5% CO_2/95% humidified air at 37°C. Following 24 h, the media was removed and cells were incubated with 50 μL of fluorescent probe (in PBS Mg (Sigma)) for 30 min, in the dark at 37°C, 5% CO_2/95% humidified air. The probe was removed, fresh PBS was added and the fluorescence was immediately read using a FLx800 Fluorescence microplate reader (Mason Technology, Dublin, Ireland). Fluorescence values were subsequently normalised to cell number using the crystal violet assay.
